# The Estimation of Blood Paramagnetic Center Changes during Burns Management with Biodegradable Propolis-Nanofiber Dressing

**DOI:** 10.1155/2020/3675603

**Published:** 2020-06-28

**Authors:** Pawel Olczyk, Katarzyna Komosinska-Vassev, Ryszard Krzyminiewski, Janusz Kasperczyk, Pawel Ramos, Bernadeta Dobosz, Olgierd Batoryna, Jerzy Stojko, Mateusz Stojko, Diana Ivanova, Krystyna Olczyk, Barbara Pilawa

**Affiliations:** ^1^Department of Community Pharmacy, Faculty of Pharmaceutical Sciences in Sosnowiec, Medical University of Silesia in Katowice, Sosnowiec, Poland; ^2^Department of Clinical Chemistry and Laboratory Diagnostics, Faculty of Pharmaceutical Sciences in Sosnowiec, Medical University of Silesia in Katowice, Sosnowiec, Poland; ^3^Medical Physics Division, Faculty of Physics, Adam Mickiewicz University, Poznan, Poland; ^4^Centre of Polymer and Carbon Materials, Polish Academy of Sciences, Zabrze, Poland; ^5^Department of Biopharmacy, School of Pharmacy with the Division of Laboratory Medicine in Sosnowiec, Medical University of Silesia in Katowice, Sosnowiec, Poland; ^6^Department of Biophysics, Faculty of Pharmaceutical Sciences in Sosnowiec, Medical University of Silesia in Katowice, Sosnowiec, Poland; ^7^Center of Experimental Medicine, Medics 4, Faculty of Medicine in Katowice, Medical University of Silesia in Katowice, Katowice, Poland; ^8^Department of Biochemistry, Molecular Medicine and Nutrigenomics, The Faculty of Pharmacy, Medical University of Varna, Varna, Bulgaria

## Abstract

The evolution of the paramagnetic center system in blood during the healing of skin burn wounds dressed with a biodegradable apitherapeutic nanofiber dressing was examined. The aim of this study was to determine the changes in paramagnetic centers in blood during the influence of apitherapeutic nanofiber dressings on the healing process. The blood samples were tested before burn infliction (day 0) and, respectively, on the 10^th^ and 21^st^ days of the experiment. Paramagnetic centers in the blood of the pig used as the model animal were examined with an X-band (9.3 GHz) electron paramagnetic resonance spectroscopy. The EPR spectra were measured with Bruker spectrometer at 230 K with a modulation frequency of 100 kHz. The EPR lines of the high spin Fe^3+^ in methemoglobin, high spin Fe^3+^ in transferrin, Cu^2+^ in ceruloplasmin, and free radicals were observed in the multicomponent spectra of blood. For the application of the apitherapeutic nanofiber dressing, the amplitudes of the EPR signals of Fe^3+^ in methemoglobin were similar up to 10 days. For the experiment with the apitherapeutic formulation, the heights of EPR signals of Fe^3+^ in transferrin were lower after 10 days and 21 days of therapy, compared to day 0. For the application of the apitherapeutic formulation the signals of Cu^2+^ in ceruloplasmin and free radicals, strongly decreased after 10 days of therapy, and after 21 days it increased to the initial values characteristic for day 0. The apitherapeutic formulation caused that after 21 days the EPR spectrum of Cu^2+^ in ceruloplasmin and free radicals was considerably high. The apitherapeutic formulation interaction after 10 days and after 21 days of therapy resulted in the low EPR lines of Fe^3+^ in methemoglobin. EPR spectra of blood may be useful for presentation of the changes in its paramagnetic centers during the healing process of the burn wounds.

## 1. Introduction

Propolis represents a complexed, natural raw material, produced, in the region of Eastern Europe by a honeybee, from balsamic substances obtained from buds of, e.g., poplar, birch, willow, alder and chestnut, and resins—among others—possessed form damaged parts of the mentioned trees [1, 2]. Bee glue, however, also contains waxes, essential and aromatic oils, pollen, feathers, dust, bee glandular secretion, and the fragments of beehives [3, 4]. For the first time, Marcucci [5] and Bankova et al. [6] have registered over 300 known substances in discussed unique natural material. The chemical compounds—encompassing the flavonoids, terpenes, and phenolics considered as bioactive markers of propolis are responsible for the pharmaceutical effects including anti-inflammatory, antimicrobial, antitumor, antiulcer and anti-HIV, antioxidant, and immunomodulatory activities [7–9]. However, the most important, from the point of view of medicine and pharmacy, is the regenerative action of propolis, which in the course of healing effectively stimulates the expression of the vascular endothelial growth factor and markedly enhances the phenomenon of cellular proliferation through the growth of H3 histone [10–14]. Propolis also enhances the burned tissue repair by stimulation of the wound matrix GAGs (CS/DS and HA) accumulation responsible for granulation, tissue growth, and wound closure [15]. Propolis modulates VN, LN, and HS/HP metabolism, leading to the better regulation of the primary wound healing cellular events, i.e., epidermal cell and keratinocyte migration and proliferation as well as fibroblast activation providing reepithelization and wound closure [16, 17]. Propolis regulates the expression and degradation of collagens types I and III in wound matrix and creates favorable biochemical environment supporting reepithelization [18]. One of the factors determining the corrective action of propolis is its antioxidant potential [19]. Despite the knowledge indicating that propolis inhibits ROS production, blocks peroxidation of LDL, and nitration of proteins, it also enhances the NOS expression and alleviates the NADPH oxidase (NOX) activity. Moreover, bee glue downregulates the damage of the DNA in cultured fibroblasts stimulated by the hydrogen peroxide (H_2_O_2_), inhibits macrophage apoptosis influencing on glutathione (GSH) and the tumor necrosis factors/nuclear factor kappa B (TNF/NF-*κ*B) pathway [20]. In this work, the unique application of electron paramagnetic resonance (EPR) spectroscopy to determine paramagnetic centers existing in blood samples taken from the experimental animals, upon therapy by an innovative biodegradable apitherapeutic nanofiber dressing, was performed. The mentioned formulation, obtained using the technique of electrospinning, containing an apitherapeutic agent, was intended for the regeneration of complicated skin wounds. The aforementioned nanofiber dressing creates the favorable environment of the wound, stimulating also reepithelialization, angiogenesis, and biosynthesis of connective tissue components, simultaneously enabling gas exchange between the wound and the environment. This innovation is the subject matter of a patent specification—“P.425636”, and it consists in incorporating a natural raw material with proven antioxidative, anti-inflammatory, immunomodulating, antiviral, antineoplastic, antibacterial, antifungal properties, not to mention the fact that it also stimulates the phenomenon of reepithelialization, and it efficiently limits the recovery time of tissue damage. Free radicals contribute to controlling the healing process at the cellular level; however, their precise role in burn wound repair is still little explored. Thus, the different types of paramagnetic centers in blood were searched. The low temperatures were used to detect iron, copper, and free radicals' signals.

## 2. Experimental

### 2.1. Tissue Material

16-week-old, domestic pig was chosen for the evaluation of wound repair because of many similarities between pig and human skin. Eighteen contact burn wounds were inflicted on the right and left flanks of the pig body, according to the methods of Hoekstra et al. [21] and Brans et al. [22]. The experimental animal was housed according to the Good Laboratory Practice (GLP) Standards of Polish Veterinary Law. Blood samples, in three replications, were taken before wounds infliction (day 0) and on postburn days 10^th^ and 21^st^. The procedure of collection of blood samples for laboratory analysis was based on a marginal ear vein cannulation. After cleaning the surface of the ear, an injection needle was inserted into the lumen of the vessel for sample collection. This procedure was repeated at each collection point [23]. After collection, the blood was stored at low temperature (-70°C). Thermal injuries were protected with nanofiber apitherapeutic dressings. The experimental protocol was accepted by the Ethics Committee of the Medical University of Silesia in Katowice, Poland (LKE-111/2014).

### 2.2. Biodegradable Nano-Nonwoven Dressings

Nano-nonwoven wound dressings are made with an electrospinning method using poly(lactide-co-glycolide) containing 85 mole-% of lactidyl and 15 mole-% of glycolidyl comonomer units (PLGA 85 : 15) [24]. 6 wt-% concentration of the polymer in solution and 1,1,1,3,3,3-hexafluoro-2-propanol as a solvent is used. The electrospinning process consists in producing the polymer fibers in the electric field between the collector with negative electric potential and the spinning nozzle, to which the positive electric potential is applied [25]. The potential difference is adjusted to 27 kV. The distance between the electrodes was set to 15 cm. To obtain the nonwoven mat, 22 ml of the solution is dosed at a rate of 1.5 ml/h [26]. Nonwoven wound dressings containing propolis [4] have been made with an electrospinning method using poly(lactide-co-glycolide) containing: 85 mole-% of lactidyl and 15 mole-% of glycolidyl comonomer units (PLGA 85 : 15) [24, 27]. A solution of propolis is introduced to the polymer solution and mixed until a homogeneous mixture is obtained. The propolis content is 5 wt-% and 10 wt-%, respectively, to the polymer used. 6 wt-% concentration of the polymer and propolis mixture in a solution and 1,1,1,3,3,3-hexafluoro-2-propanol as a solvent is used. The electrospinning process consists in producing the polymer fibers in the electric field between the collector with negative electric potential and a spinning nozzle, to which the positive electric potential is applied [25]. The potential difference is adjusted to 27 kV. The distance between the electrodes was set to 15 cm. To obtain the nonwoven mat, 22 ml of solution is dosed at a rate of 1.5 ml/h [26]. The blood samples are placed in the thin-walled tubes with an external diameter of 3 mm and the EPR spectra are measured.

### 2.3. EPR Measurements

Paramagnetic centers in blood samples were examined by the use of electron paramagnetic resonance spectroscopy (EPR) at low temperature equaled 230 K. This spectroscopic method was chosen, because of the direct experimental procedures providing information about molecules containing unpaired electrons [28]. Energy levels of unpaired electrons in the paramagnetic centers located in the magnetic field are split. Unpaired electrons absorb microwaves of the suitable frequencies, which bring the energy adequate to the distances between the energy levels, and the unpaired electrons go to the higher energy levels. On the way of relaxation processes, the unpaired electrons of the tested paramagnetic centers go back to the lower levels. The absorbed energy of microwaves is measured as the resonance curves, which parameters give information about paramagnetic centers [28, 29]. The important advantages of the EPR method are its nondestructive nature relative to the examined substances [28, 29].

The electron paramagnetic resonance spectra were measured as the first-derivative curves by an X-band (9.3 GHz) EPR spectrometer produced by Bruker (USA). Magnetic modulation of 100 kHz was used. The low microwave power of 7.9 mW was used to avoid microwave saturation of EPR lines. The cryogenic system to low-temperature measurements of Bruker (USA) was used. For the studied blood samples, the following parameters of EPR spectra were analyzed: g-factors and amplitudes (A). Amplitudes (A) of the EPR lines increase with increasing of the amount of paramagnetic centers in the samples [28]. g-Factor depends on the type of paramagnetic centers, which are responsible for the EPR line. g-Values will be calculated from resonance condition according to the formula [28, 29]:

(1) $$\begin{array}{c} \text{g}=\text{h}\nu /\mu_{\text{B}}{\text{B}}_{\text{r}}. \end{array}$$

where h is Planck constant, *ν* is microwave frequency, *μ*_B_ is Bohr magneton, and B_r_ is resonance magnetic field.

The expressions of “h*ν*” and “g*μ*_B_B_r_” mean the energy of microwaves and the distance between the energy levels, respectively [28]. Microwave frequency (*ν*) was directly measured by the Bruker recorder. The B_r_ values were determined from the electron paramagnetic resonance lines. In the present paper, we followed the same EPR method of examination of paramagnetic centers in the blood as in the previous our work [30] concerning the low-temperature electron paramagnetic resonance application.

## 3. Results and Discussion

The present study is a continuation of our previous experimental studies concerning the usefulness of a new low-temperature electron paramagnetic resonance technique for monitoring molecular complexes containing iron Fe3+ (methemoglobin, transferrin) or copper Cu2+ ions (ceruloplasmin) and free radicals in the blood during healing of burn wounds treated with biodegradable dressings containing poly(lactide-co-glycolide). The obtained results indicated that a more complete assessment of biochemical changes taking place in the process of repairing tissue damage after burn insult is needed, especially in the scope of mechanisms regulating the metabolism of iron and copper ion complexes as well as free radicals in the blood. Followed by EPR technic described in our previous work [30], in this study, we examined the multicomponent electron paramagnetic resonance spectra of the blood during healing taken from the experimental animal, obtained after 1^st^, 10^th^, and 21^st^ days of burn treatment with a previously used biodegradable dressings additionally containing pharmacologically active substance of natural origin—propolis.

The evolution of the healing process undergoing three overlapping phases, (1) hemostasis and inflammation, (2) proliferation, and (3) remodelling, has been summarized and presented in Table 1. The effects of management with biodegradable propolis-nanofiber dressing on each would healing stage were also included in this table (Table 1).

**Figure 1:**
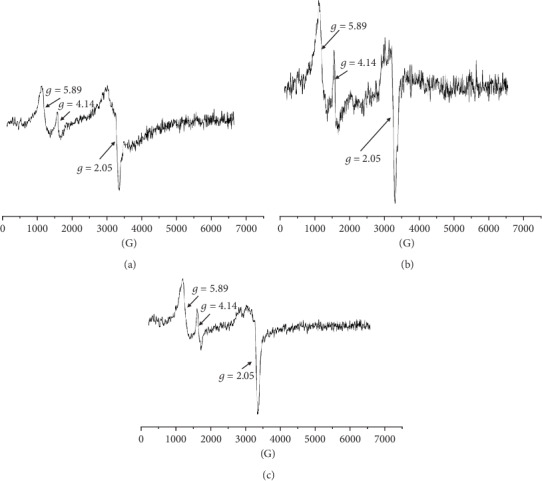
The first derivative EPR spectrum of paramagnetic centers: the high spin Fe^3+^ in methemoglobin (line I), high spin Fe^3+^ in transferrin (line II), Cu^2+^ in ceruloplasmin and free radicals (line III), in blood for skin (a) before burn infliction (day 0) and for burned wounds treated with the propolis nanofiber dressing at: (b) 10 and (c) 21 days of therapy. B—magnetic induction.

**Figure 2:**
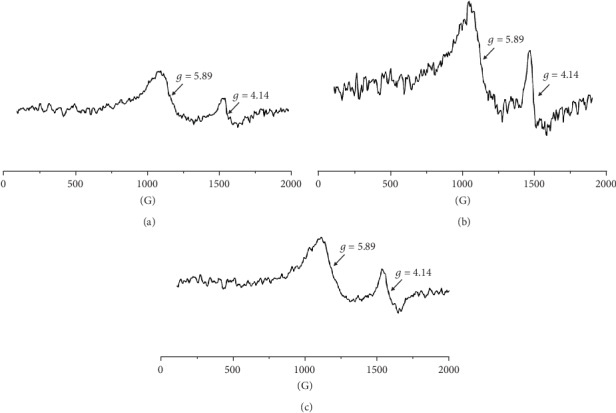
The EPR lines of the high spin Fe^3+^ in methemoglobin (line I) and high spin Fe^3+^ in transferrin (line II), in blood for skin (a) before burn infliction (day 0) and for burned wounds treated with the propolis nanofiber dressing on: (b) 10 and (c) 21 days of therapy. B—magnetic induction.

For all the tested blood samples, the complex EPR spectra were measured [28, 29]. The EPR spectra of paramagnetic centers in blood for skin before inflicting thermal damages (day 0) and for burned wounds treated with the propolis nanofiber dressing 10 and 21 days of therapy were shown in Figures 1(a)–1(c), respectively. Three lines (signed as I, II, and III) were observed. The line III was a superposition of two signals. Taking into account the earlier results of EPR studies of blood [31], these lines come from high spin Fe^3+^ in methemoglobin (line I), high spin Fe^3+^ in transferrin (line II), and Cu^2+^ in ceruloplasmin and free radicals (line III). The low field signals (line I, line II) of the EPR spectra of blood in the experiment with the dressing containing propolis for day 0 (before burn infliction), 10 days, and 21 days of therapy were presented in Figures 2(a)–2(c), respectively.

**Figure 3:**
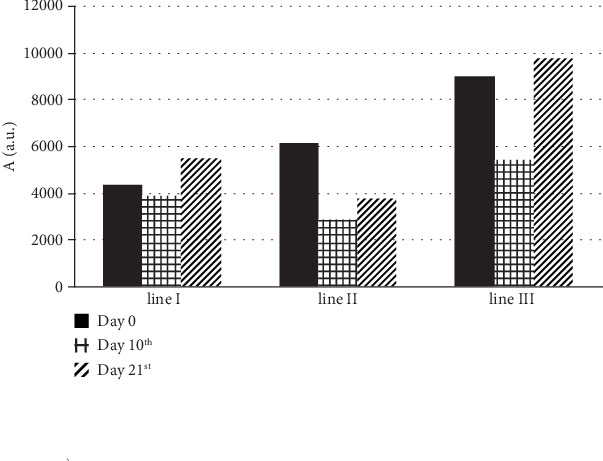
The influence of time of therapy for the amplitudes (A) of the high spin Fe^3+^ in methemoglobin (line I), high spin Fe^3+^ in transferrin (line II), and Cu^2+^ in ceruloplasmin and free radicals (line III), in blood for burned skin wounds treated with the propolis. The data, obtained on day 0 (before burn infliction) and, respectively, 10 and 21 days of therapy, were compared.

The concentration of the individual types of paramagnetic centers in blood changed with time of therapy for propolis nanofiber dressing. This effect was observed with changes in the individual components of the multicomponent EPR spectra of blood. Their heights depended on the time of therapy with the apitherapeutic formulation. The influence of time of therapy on the amplitudes (A) of the high spin Fe^3+^ in methemoglobin (line I), high spin Fe^3+^ in transferrin (line II), and Cu^2+^ in ceruloplasmin and free radicals (line III), in blood for burned skin wounds treated with the mentioned biodegradable dressing, was presented in Figure 3.

For the propolis nanofiber dressing, the amplitudes (A) of the EPR signals of Fe^3+^ in methemoglobin (line I) were similar up to 10 days, and the amplitude (A) of the (line I) for the blood after 10 days of therapy was only slightly lower than the amplitude (A) of this line measured before burn infliction (day 0) (Figure 3). Its value increased after 21 days of therapy (Figure 3). For the experiment with the propolis dressing, the heights of EPR signals of Fe^3+^ in transferrin (line II) were lower after 10 days and 21 days of therapy, compared to day 0 (Figure 3). For the application of the apitherapeutic formulation, the signals of Cu^2+^ in ceruloplasmin and free radicals (line III) strongly decreased after 10 days of therapy, and after 21 days of therapy it increased slightly over the initial value characteristic for the day before burn infliction (Figure 3). The comparison of the multicomponent EPR spectra of blood may be useful for the presentation of the changes in its paramagnetic centers during the healing process of the burned wounds. Repair of the damaged tissue represents the “fundamental” response to injury, including the replacement of damaged structures with a living tissue that restores the integrity of the skin [32]. In order to modify the wound healing, throughout the reduction or elimination of scarring or to achieve more effective restoration of normal tissue, it is necessary to understand the mechanism of oxidative stress influence on all of the stages of the regeneration process [33–35].

The first phase of wound healing begins immediately upon injury and is dedicated to hemostasis and the formation of a provisional wound matrix. In response to tissue injury, inflammatory cells, including neutrophils, monocytes, and macrophages, are recruited to wounded tissue. Free radicals released mainly from activated neutrophils contribute to local tissue impairment causing oxidative damage of proteins, lipids, DNA, and RNA [35]. In this way, free radicals are participants in local damage following thermal injury. The acute inflammatory response is followed by the proliferative phase of wound healing, when the wound is rebuilt with new granulation tissue made up of collagen and other extracellular matrix components. The main objective of the repair phase is to achieve protection of the wound's surface via the formation of granulation tissue and a new epithelial cover and to restore the vascular network to nourish the new tissues. It should be noted that granulation is an oxygen-dependent process [36–38].

Repair of the skin ends with a remodeling phase, which results in would contraction and scar tissue formation. Collagen is remodeled from type III to type I. Cross-linking of collagen reduces scar thickness and makes the skin area of the wound stronger. Adequate level of oxygen is absolutely essential for the cross-linking process [39–41].

The proposed concept of the role of free radicals in each of the wound healing stage has important implications in burn management, indicating that compounds with antioxidant potential, including propolis, can improve the healing of thermal burns.

Except for the formation of free radicals, injury elicits the acute phase response, which increases in hepatic synthesis of proteins, including among others, ceruloplasmin and transferrin. Taking part in the oxidative stress transferrin, ceruloplasmin, and methemoglobin are also involved in iron metabolism [42]. Previously described metal—a vital cofactor for proteins and enzymes involved in energy metabolism, respiration, DNA synthesis, cell cycle arrest, and apoptosis—during oxidative stress processes, acts as a transition metal, which exists in two stable states, Fe^2+^ (electron donor) and Fe^3+^ (electron acceptor) [43]. In the course of wound healing, the mentioned metal is postulated to play a beneficial role in collagen synthesis, while the iron deficiency results in an impaired T cell and phagocyte function during the inflammatory phase, resulting in a subsequent decreased tensile strength [44]. Moreover, during the earliest phase of wound repair, the level of the another estimated indicatory molecule—transferrin (Tf) can be elevated, in response to infection and inflammation, therefore the concentration of Tf usually increases. What is of particular interest, Tf represents the bacteria's growth inhibition factor—essential to prevent tissue damage [45, 46]. Therefore, reduced transferrin amplitude on the 10^th^ day of the experiment may be caused by the use of iron in the course of the Fenton reaction during the acute phase of wound healing [43]. Last but not least, the increase in transferrin amplitude estimated in blood samples, collected on the 21^st^ day of burns management with the novel apitherapeutic dressing, may be connected with the Tf's influence on growth and the formation of extracellular matrix, necessary for the proper regulation of biosynthesis phase of the repair process, manifested by stimulation of the collagen biosynthesis [47] and enhancement of accumulation of dermatan/chondroitin sulfate proteoglycans [48]. Another protein of particular importance not only in case of oxidative stress but also in relation to iron metabolism is methemoglobin (MetHb). The mentioned molecule, containing ferric Fe^3+^ iron rather than ferrous Fe^2+^ one, characterised by the decreased ability to bind oxygen, is reported to be reduced in blood after propolis application [49]. The last-mentioned phenomenon seems to be important due to an oxidative damage of haemoglobin resulting in Heinz body formation. Therefore, methemoglobinemia represents a widely used indicator of oxidant damage of red blood cells, determining functional disturbances of membrane and cytoplasmic structures [50]. Furthermore, according to Moreira et al. [51], propolis was also shown to inhibit the production of methemoglobin under the action of hydrogen peroxide—released by phagocytes to clear tissue debris or kill the colonizing microorganisms—enhancing the hyaluronan depolymerization [52]. The increase in methemoglobin amplitude found on the final day of the study, remaining in contradiction with the one stated by Ercis et al. [49], may paradoxically indicate that the time of observation of the impact of the innovative propolis nanofibers on the amplitude of methemoglobin in the sera of experimental animals was too short. Therefore, future observations of the effects of propolis dressing will include an extended period that will probably reveal the overall effect of the proposed propolis formulation on the amplitude of methemoglobin in the blood of experimental animals, qualified for the experimental design of burn wound healing. Moreover, during the abovementioned phenomenon, the arising wound bed matrix can be protected by the copper and their transporting, acute phase, protein - ceruloplasmin responsible for the catalizing the ferrous ion into the ferric one [53]. Moreover, ceruloplasmin and copper are crucial for lysyl oxidase and the extracellular cross-linking and maturation of collagen and elastin [54]. Ceruloplasmin—increasing during wound healing—participates in the phospholipid synthesis, necessary for cell membrane creation in the regenerating matrix [52]. However, in the face of oxidative stress reactive oxygen species, including hydrogen peroxide, disrupted copper binding to ceruloplasmin causes a release of the ion responsible for the promotion of oxidative pathology [55]. Therefore, in the course of wound healing particular importance seems to be gained by, the activity of propolis reported to increase the activity of the ceruloplasmin [56, 57]. The last observation seems to confirm the results of the present study experiment, in the course of which, after a previous fall recorded on the 10th day, along with the 21st day of experience, propolis dressing stimulates the increase in ceruloplasmin amplitude, which reaches the values observed at the beginning of the experiment. The evaluation of these molecules, using a unique EPR method, in blood samples of experimental animals may serve to assess the healing effectiveness of the used for the first time, in the experimental burn wounds healing model, the innovative biodegradable dressing containing propolis.

## 4. Conclusions

The preformed electron paramagnetic resonance studies of blood from the organisms in the examples of the skin of burned wounds treated with biodegradable, propolis containing, dressing have pointed out that:

1. The high spin Fe^3+^ in methemoglobin, high spin Fe^3+^ in transferrin, and Cu^2+^ in ceruloplasmin and free radicals exist in all the tested blood samples, and they are responsible for their multicomponent EPR spectra
2. The amplitudes of the EPR lines, which reflected the concentrations of high spin Fe^3+^ in methemoglobin, high spin Fe^3+^ in transferrin, and Cu^2+^ in ceruloplasmin and free radicals in the blood, depends on the time of therapy
3. Paramagnetic centers and free radicals' changes indicate a favorable effect of innovative biodegradable apitherapeutic dressings on burns regeneration, suggesting a pluripotent multifaceted influence of propolis on the prooxidative/antioxidative balance changes whose components serve a fundamental function in the repair of tissue damages

## Figures and Tables

**Table 1:** Effects of management with biodegradable propolis-nanofiber dressing on each wound healing stage.

Hemostasis and inflammatory phase	Proliferative phase	Tissue remodelling phase
(i) Early phase of inflammation begins when the wound develops, lasts 4-6 days (ii) Trauma causes peripheral blood platelets and neutrophils to migrate to the injury, forming a fibrin clot to end bleeding, and the inflammatory stage of the healing cascade begins (iii) Increased free radical formation appears as a result of the local tissue damage and a systemic inflammatory response	(i) Lasts another 3-10 days postinjury (ii) Fibroblasts, endothelial cells and epithelial cells migrate into the wound bed (iii) Granulation tissue formation—fibroblasts proliferate and synthesize new components of extracellular matrix (iv) Angiogenesis—new blood vessels carry oxygen and nutrients necessary for the metabolism and growth of cells, and confer to the granulation tissue its characteristic red, granular appearance (v) Covering the wound (reepithelialization)—epithelial cells migrate from the wound bed or margins	(i) Begins about 21 days postinjury and can continue for a year (ii) Wound contraction and scar tissue formation occurs (iii) Collagen is remodeled from type III to type I cross-linking of collagen reduces scar thickness and makes the skin area of the wound stronger (iv) The wound fully closes (v) The cells that had been used to repair the wound but which are no longer needed are removed by apoptosis

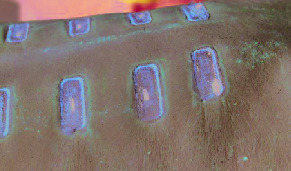	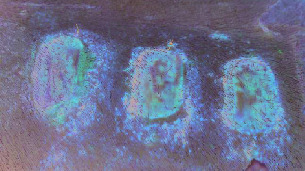	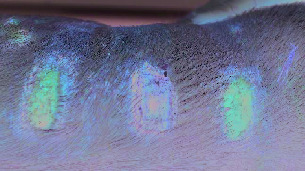
Burns management with PLGA 85/15 dressing with 5% propolis Day 0 (i) Necrosis at the burn induction site and in the wound area from 3 to 15 mm from the edge (ii) Intense redness and swelling around the necrotic area (iii) Exudation, visible tissue at the burn site carbonization	Burns management with PLGA 85/15 dressing with 5% propolis 10^th^ day (i) A thin scab not completely covering the wound area (ii) The wound is increasingly covered with a thin layer of epidermis (iii) Bristle growth all over the wound area (iv) No redness around the wound area	Burns management with PLGA 85/15 dressing with 5% propolis 21^st^ day (i) The wound covered with pink epidermis, (ii) Significant reduction in the wound surface (iii) Small fragments of the elastic and protruding scab (iv) Regrowth bristles visible at the burn induction site (v) No edema and inflammation around the wound

## Data Availability

The WINEPR data used to support the findings of this study have been deposited in the computer that supports an EPR spectrometer produced by Bruker (USA) repository, Medical Physics Division, Faculty of Physics, Adam Mickiewicz University, Poznan, Poland. The contact person is Professor Ryszard Krzyminiewski (rku@amu.edu.pl). The electrospinning method data used to obtain samples of biodegradable, nonwoven dressings are patentprotected and so cannot be made freely available. Request for access to these data should be made to Professor Janusz Kasperczyk (jkasperczyk@cmpw-pan.edu.pl) or Mateusz Stojko (mstojko@cmpw-pan.edu.pl).
